# Speciation in the dark: diversification and biogeography of the deep‐sea gastropod genus *Scaphander* in the Atlantic Ocean

**DOI:** 10.1111/jbi.12471

**Published:** 2015-01-30

**Authors:** Mari H. Eilertsen, Manuel António E. Malaquias

**Affiliations:** ^1^ Marine Biodiversity Research GroupDepartment of BiologyUniversity of Bergen5006BergenNorway; ^2^ Phylogenetic Systematics and Evolution Research GroupDepartment of Natural HistoryUniversity Museum of BergenUniversity of Bergen5020BergenNorway

**Keywords:** Allopatric speciation, amphi‐Atlantic, Cephalaspidea, dispersal, diversification pulse, Heterobranchia, marine biogeography, Mollusca, Tethyan vicariance

## Abstract

**Aim:**

The aim of this work was to improve understanding about the mode, geography and tempo of diversification in deep‐sea organisms, using a time‐calibrated molecular phylogeny of the heterobranch gastropod genus *Scaphander*.

**Location:**

Atlantic and Indo‐West Pacific (IWP) oceans.

**Methods:**

Two mitochondrial gene markers (*COI* and *16S*) and one nuclear ribosomal gene (*28S*) from six Atlantic species of *Scaphander*, and four IWP species were used to generate a multilocus phylogenetic hypothesis using uncorrelated relaxed‐clock Bayesian methods implemented in beast and calibrated with the first occurrence of *Scaphander* in the fossil record (58.7–55.8 Ma).

**Results:**

Two main clades were supported: clade A, with sister relationships between species and subclades from the Atlantic and IWP; and clade B, with two western Atlantic sister species. Our estimates indicate that the two earliest divergences in clade A occurred between the middle Eocene and late Miocene and the most recent speciation occurred within the middle Miocene to Pleistocene. The divergence between the two western Atlantic species in clade B was estimated at late Oligocene–Pliocene.

**Main conclusions:**

The prevailing mode of speciation in *Scaphander* was allopatric, but one possible case of sympatric speciation was detected between two western Atlantic species. Sister relationships between IWP and Atlantic lineages suggest the occurrence both of vicariance events caused by the closure of the Tethyan Seaway and of dispersal between the two ocean basins, probably around South Africa during episodic disruptions of the deep‐sea regional current system caused by glacial–interglacial cycles. Cladogenetic estimates do not support comparatively older diversification of deep‐sea faunas, but corroborate the hypothesis of a pulse of diversification centred in the Oligocene and Miocene epochs. Amphi‐Atlantic species were found to occur at deeper depths (bathyal–abyssal) and we hypothesize that trans‐Atlantic connectivity is maintained by dispersal between neighbouring reproductive populations inhabiting the abyssal sea floor and by dispersal across the shelf and slope of Arctic and sub‐Arctic regions.

## Introduction

Little is known about the biogeography and speciation mechanisms of deep‐sea (> 200 m deep) organisms (Thistle, 2003). This is in sharp contrast with shallow‐water faunas, where the advent of molecular phylogenetic methods during the last 20 years has led to a profusion of studies that improve our understanding of the origins of species and patterns of diversity (e.g. Williams & Reid, 2004; Williams & Duda, 2008; Malaquias & Reid, 2009; Bowen *et al*., 2013). Some factors commonly invoked to explain diversification in shallow water (e.g. sea‐level fluctuations, variation in water temperature) are unlikely to have the same impact in the deep sea, but a common period of diversification for both shallow and deep‐water faunas was recently suggested around the Oligocene and Miocene epochs, probably influenced by global warming, high levels of tectonic activity and changes in oceanic currents (Williams & Duda, 2008; Cabezas *et al*., 2012; Williams *et al*., 2013).

At a global scale, the effects of major tectonic events on the biogeography and diversification of shallow‐water marine faunas are well documented, and include the opening of the Drake Passage and establishment of the Antarctic Circumpolar Current (Beu *et al*., 1997), the closure of the Tethys Sea (Rögl, 1998), the opening of the Bering Strait (Marincovich & Gladenkov, 1999) and the uplift of the Isthmus of Panama (Lessios, 2008). Nevertheless, how these same events affected deep‐water fauna remains largely unknown.

The traditional view has been that deep‐sea species have large biogeographical ranges due to the apparent lack of barriers to dispersal and homogeneous environment (McClain & Hardy, 2010). The notion of the deep sea as a continuous and homogeneous ecosystem where diversity and biogeographical patterns are mostly controlled by biological interactions such as competition (the stability–time hypothesis; Sanders, 1968), has been challenged by the discoveries of chemosynthetic habitats [hydrothermal vents (Lonsdale, 1977), cold seeps (Paull *et al*., 1984) and whale‐falls (Smith *et al*., 1989)], seasonality (Billett *et al*., 1983) and deep‐sea storms (Hollister & McCave, 1984). Today, the deep sea is regarded as a patchy environment of great heterogeneity, created by disturbances at different scales, generating a mosaic of communities at different stages of succession (temporal mosaic hypothesis; Grassle & Sanders, 1973; Levin *et al*., 2001; Rex & Etter, 2010). These two hypotheses are mostly based on contemporaneous ecological factors, however, and do not take large‐scale temporal effects or an evolutionary perspective into account.

There are very few molecular phylogenies of benthic deep‐sea clades with complete (or even near‐complete) taxon sampling, and the few available works have a regional focus (e.g. Puillandre *et al*., 2010; Cabezas *et al*., 2012; Dueñas *et al*., 2014) or are concerned with the origins of major deep‐sea clades (genus‐level or higher; e.g. Strugnell *et al*., 2008; Lins *et al*., 2012; Williams *et al*., 2013; Corrigan *et al*., 2014). More attention has been paid to the genetic diversity of broadly distributed species, such as the bivalves *Deminucula atacellana* and *Ledella ultima* (Zardus *et al*., 2006; Etter *et al*., 2011) or the amphipod *Eurythenes gryllus* (Havermans *et al*., 2013). Differences in geographical ranges between organisms that inhabit the bathyal zone (*c*. 1000–3000 m) and those that inhabit the abyssal plains (*c*. 4000 m) have been recognized, and studies on deep‐sea bivalves and gastropods have shown that abyssal species have larger biogeographical ranges than bathyal species (Etter & Rex, 1990; Allen & Sanders, 1996; Etter *et al*., 2005, 2011; Zardus *et al*., 2006).

Despite the fact that evidence is slowly accumulating, biogeographical inferences on deep‐sea faunas are still hampered by three main factors.

1. Taxonomic impediment. Many deep‐sea species with broad geographical ranges have been identified based on similarities of their external morphology only (e.g. Allen & Sanders, 1996; Allen, 2008; Miljutin *et al*., 2010; Menzel *et al*., 2011), and there is growing recognition of cryptic speciation in the marine realm (Carmona *et al*., 2011; Claremont *et al*., 2011).
2. Sampling bias. It is acknowledged that very little of the deep sea has actually been sampled (less than 0.01%; Ramirez‐Llodra *et al*., 2010), and that sampling is uneven across ocean basins. For example, the South Atlantic is much less sampled than the North Atlantic (McClain & Hardy, 2010).
3. Sampling constraints. Deep‐sea sampling is expensive and the amount of material suitable for DNA analysis available in worldwide research institutions is very low. This is especially limiting for phylogenetic analyses where high levels of geographical and taxon coverage is desirable.

*Scaphander* is a globally distributed genus of predominantly deep‐sea cephalaspidean gastropods (Eilertsen & Malaquias, 2013a). Eight species of *Scaphander* are recognized in the Atlantic Ocean, and these are well supported based on shell morphology, internal anatomy (including characters of the male reproductive system) and reciprocal monophyly in a molecular phylogenetic tree (Eilertsen & Malaquias, 2013a). Because of its large, strong shell, *Scaphander* is relatively well documented in the fossil record. Palaeocene fossils are known from Europe, the USA and the east and west coasts of Argentina, with the oldest fossils that can confidently be identified as *Scaphander* dating from the late Palaeocene (Thanetian, 58.7–55.8 Ma) of Poland (Krach, 1963) and California (Weaver, 1949; Schoellhamer *et al*., 1981).

Our main goal is to contribute to a better understanding of the mode, causes and tempo of diversification of deep‐sea organisms, with an emphasis on the Atlantic Ocean and using *Scaphander* snails as our study group. We will produce a time‐calibrated molecular phylogeny to infer relationships and estimate the age of divergence between species. This phylogeny, together with knowledge about the geographical distributions of species, will be used to infer the prevalent geographical mode of speciation and test the hypothesis that the Oligocene and Miocene epochs correspond to a period of major diversification of deep‐sea fauna. We will test the prediction that, in deep‐sea groups, speciation between Atlantic and Indo‐Pacific lineages caused by the closure of the Tethyan Seaway is expected to pre‐date the diversification times of shallow‐water species. We will also assess whether species of *Scaphander* with deeper bathymetric ranges have broader geographical distributions, and then discuss the possible causes.

## Materials and methods

### Taxon sampling, geography and bathymetric distributions

Our set of specimens for molecular study was assembled from natural history museums and from fieldwork along the coast of Norway (Table 1). Geographical and bathymetric distributions of all Atlantic species of *Scaphander* were taken from Eilertsen & Malaquias (2013a). Only bathymetric records of live specimens were considered to be reliable, and therefore the bathymetric distribution of *S. gracilis* is not included here, because this species is only known from empty shells. Geographical and bathymetric distributions of Indo‐Pacific species were not plotted in detail because there is no comprehensive revision available for these species and they are not the focus of the present study.

**Table 1:** List of specimens used for molecular phylogenetic analysis including sampling localities and voucher numbers. Sequences labelled with an asterisk (*) were generated for the present study, the remaining sequences were downloaded from GenBank. Abbreviations: MCZ, Museum of Comparative Zoology, Harvard University, Boston, MA, USA; MNHN, Muséum national d'Histoire naturelle, Paris, France; MZSP, Museu de Zoologia da Universidade de São Paulo, Brazil; NHMUK, Natural History Museum, London, UK; RMNH, National Museum of Natural History (Naturalis), Leiden, The Netherlands; USNM, National Museum of Natural History, Smithsonian Institution, Washington DC, USA; ZMBN, Natural History Collections, University Museum of Bergen, Norway

Species	Specimen	Locality	Voucher number	*COI*	*16S*	*28S*
*Scaphander lignarius*	1	Cádiz, Spain	ZMBN 87998	KC731431*	KC351524	KC351543
	2	Bergen, Norway	ZMBN 87999	KC351562	KC351525	KC351544
	19	Barcelona, Spain	MCZ 371884	KC351561	KC351522	
	37	Bergen, Norway	ZMBN 88000	KC351563	KC351526	KC351545
	51	Lofoten, Norway	ZMBN 88001	KC351564	KC351527	KC731432*
	GB1	Algarve, Portugal	NHMUK 20060325	DQ974663	DQ923454	DQ927221
	GB2	Algarve, Portugal	NHMUK 20060114	DQ974664		DQ927212
	GB3	Blanes, Spain	EED‐Phy‐442			EF489372
*Scaphander punctostriatus*	3	Bergen, Norway	ZMBN 88002	KC351568	KC351532	KC351549
	4	Newfoundland, Canada	MNHN, Paris	KC351566	KC351531	KC351548
	34	Lofoten, Norway	ZMBN 88006	KC351571	KC351536	KC351553
	35	Honningsvåg, Norway	ZMBN 88005	KC351570	KC351535	KC351551
	36	Skagerrak, Denmark	ZMBN 88004	KC351569	KC351534	KC351552
	38	Hauglandsosen, Norway	ZMBN 88003	KC351567	KC351533	KC351550
*Scaphander watsoni*	15	Tampa, FL, USA	USNM 1151226	KC351575	KC351542	KC351557
	17	New Orleans, LA, USA	USNM 1151240	KC351576	KC731433*	KC351558
*Scaphander nobilis*	9	Bay of Biscay, France	MNHN, Paris		KC351530	
*Scaphander bathymophilus*	13	Azores, Portugal	RMNH unnr.	KC351559	KC351520	
	52	San Juan, Puerto Rico	MZSP 75708	KC731430*	KC351519	
*Scaphander darius*	21	Guarapari, Brazil	MZSP 29016	KC351560	KC351521	
*Scaphander mundus*	29	East of the Philippines	MNHN, IM‐2009‐4319	KC351565	KC351529	KC351547
	31	East of the Philippines	MNHN, IM‐2009‐4318	KC731429*	KC351528	KC351546
*Scaphander* sp. A	30	Grand Passage, New Caledonia	MNHN, IM‐2009‐4317	KC351572	KC351537	KC351554
*Scaphander* sp. B	32	Grand Passage, New Caledonia	MNHN, IM‐2009‐4371	KC351573	KC351538	KC351555
*Scaphander subglobosus*	33	Bohol Sea, Philippines	MNHN, IM‐2009‐4339	KC351574	KC351539	KC351556
*Sagaminopteron psychedelicum*		Kalakajoro, Madagascar	Cas**‐**Cephas3	DQ974667	KJ022787	DQ927225

### Molecular phylogenetic analyses and estimation of divergence times

Sequences of the mitochondrial genes cytochrome oxidase *c* subunit I (*COI*) and 16S rRNA (*16S*) and the nuclear gene 28S rRNA (*28S*) of *Scaphander* were obtained from GenBank (previously generated by Eilertsen & Malaquias, 2013a) and some additional sequences were produced (Table 1). DNA was extracted, amplified and sequenced according to the protocol described by Eilertsen & Malaquias (2013a), except for some specimens where amplification of *28S* failed. These were run again using LA Taq polymerase with GC buffer from TaKaRa (TaKaRa Bio, Otsu, Japan). The PCR reaction volume was 25 μL, comprising 5.35 μL ddH_2_O, 12.5 μL GC buffer, 4 μL dNTPs, 1 μL of each primer (10 μM concentration), 0.15 μL Taq and 1 μL DNA template. PCR thermal cycles were as follows: initial denaturation of 1 min at 94 °C, followed by 40 cycles with denaturation for 30 s at 94 °C, annealing for 30 s at 52 °C, and extension for 2 min at 72 °C. Final extension was 10 min at 72 °C. PCR products were cleaned and sequenced as described in Eilertsen & Malaquias (2013a).

sequencher 4.10.1 (Gene Codes, Ann Arbor, MI, USA) was used to assemble the forward and reverse strands and to assess the quality of the sequences, which were edited by careful examination of chromatograms. Sequences were checked for potential contamination using blast (Altschul *et al*., 1990) and have been deposited in GenBank (Table 1). The sequences were aligned using clustal X (Thompson *et al*., 1997) with a gap‐opening penalty of 60 and a gap‐extension penalty of 30. The single‐gene alignments were examined in bioedit (Hall, 1999), padded to equal the longest sequence and missing data at the ends were coded with question marks. Blocks of ambiguous data in the single‐gene alignments were identified and excluded using gblocks with relaxed settings (Talavera & Castresana, 2007; Kück *et al*., 2010; see Appendix S1 in Supporting Information). Pairwise uncorrected *p*‐distances were calculated using mega 5.2 (Tamura *et al*., 2011). The incongruence length difference test (ILD; Farris *et al*., 1995), implemented in paup* 4.0 b10 (Swofford, 2003) as the partition homogeneity test, was performed on the dataset with 100 replicates to test for incongruence between genetic markers. Saturation was tested for each gene and for the first, second and third codon positions of the *COI* gene, by plotting GTR pairwise distances against total substitutions (transitions + transversions). Substitution saturation analysis showed signs of saturation at the third codon position of *COI*, so two *COI*  datasets were created: one including the third position (*COI*‐A) and one excluding it (*COI*‐B).

The best‐fitting models of evolution (see Appendix S2) were selected according to the Akaike information criterion (Akaike, 1974) implemented in mrmodeltest 2.3 (Nylander, 2008). For *COI* and *16S*, the GTR+G+I model was the best model (Appendix S2), but because of statistical concerns regarding the coestimation of the gamma and invariant‐site parameters (discussed in the RaxML manual; Stamatakis, 2008) we chose to use GTR+G for these genes. Chronograms for each of the single‐gene datasets (*COI*,* 16S* and *28S*) and for a concatenated dataset of all three genes with missing data coded as question marks were produced in beast 1.8.0 (Drummond *et al*., 2007). The analyses were set up in BEAUti 1.8.0 (Drummond *et al*., 2007) with the concatenated dataset partitioned by gene, using unlinked substitution models and clock models and linked tree priors. The ingroup (all species of *Scaphander*) was defined and set to be monophyletic. The sister lineage of *Scaphander* is not known and we therefore selected as an outgroup a representative of the family Gasteropteridae (*Sagaminopteron psychedelicum*), which has been shown to be closely related to Scaphandridae (Malaquias *et al*., 2009).

A relaxed, uncorrelated lognormal clock model was selected, and substitution rates were left to be estimated (Drummond *et al*., 2006). The tree model was chosen using Bayes factor (BF) calculations comparing the Yule speciation model (Gernhard, 2008) and the birth–death model with incomplete sampling (Stadler, 2009) based on single‐run marginal likelihoods obtained by stepping‐stone (SS) sampling. The result [2 ln(BF) = 12.2] strongly favoured the birth–death model with incomplete sampling (Kass & Raftery, 1995). The prior for the proportion of taxa sampled was modelled as a normal distribution with a mean of 0.45 and a standard deviation of 0.05. The time to the most recent common ancestor (TMRCA) for *Scaphander* was given a lognormal prior with an offset of 55.8 Ma and, a mean of 1.5 Myr and a standard deviation of 1 Myr (Ho & Phillips, 2009). These settings were based on the oldest reliable fossils of *Scaphander*, which date from the late Palaeocene (55.8–58.7 Ma; Weaver, 1949; Krach, 1963; Schoellhamer *et al*., 1981). A vaguely informative prior (exponential distribution, mean 0.1) was set for the relaxed clock rates, and the remaining priors were left at the default settings (Drummond *et al*., 2007). Three independent runs were carried out and each analysis was run for 20 million generations for the single‐gene datasets, and 50 million generations for the combined dataset, with sampling every 1000 generations. The log files were examined in tracer 1.5 (Rambaut & Drummond, 2009) to ensure convergence was reached and to determine the burn‐in. The outputs were combined in logcombiner and maximum clade credibility trees created in treeannotator (Drummond & Rambaut, 2007) with a burn‐in of 10%. The resulting trees were converted to graphics in figtree 1.4.0 (Rambaut, 2012) and final adjustments were made in adobe illustrator CS6 (Adobe Systems, San Jose, CA, USA).

## Results

### DNA sequence analyses

The present dataset includes six of eight valid Atlantic species (75% of recognized diversity; Eilertsen & Malaquias, 2013a), and four Indo‐Pacific species (40% of recognized diversity; Valdés, 2008; Rosenberg *et al*., 2012; for the complete specimen list see Table 1). Seven sequences from *S. lignarius* were downloaded from GenBank and included in the dataset. The gblocks analysis excluded 10 positions from the *COI* alignment with third codon positions excluded (*COI*‐B), 27 positions from the *16S* alignment and 25 positions from the *28S* alignment (all positions excluded were at the ends of the alignments; see Appendix S1 for settings). Uncorrected *p*‐distances for *COI* (*COI*‐A, complete alignment) ranged from 0.1% to 5.9% within *Scaphander* species and 10.8–19.7% between species; however, two specimens of *S. lignarius* from Spain (specs 1 and 19) showed unusually high divergence from conspecifics (9.6–10.1%). For *16S*, uncorrected *p*‐distances ranged between 0–2% within *Scaphander* species and 0.4–10.1% between species. It is interesting to note that the two *S. lignarius* specimens from Spain (specimens 1 and 19) also showed high intraspecific divergence in *16S*, along with one specimen from Portugal (specimen GB1), with 1–2% differences from conspecifics. This may indicate some phylogeographical structure in *S. lignarius*, but it would require a much larger dataset with better geographical coverage to test this hypothesis.

The ILD test (Farris *et al*., 1995) performed on the concatenated dataset showed no incongruence between the genetic markers (*P* ≫ 0.05). Because substitution saturation analysis showed signs of saturation in the third codon position of *COI*, the alignment excluding this position (*COI*‐B) was used in the phylogenetic analyses.

### Phylogenetic hypothesis

**Figure 1:**
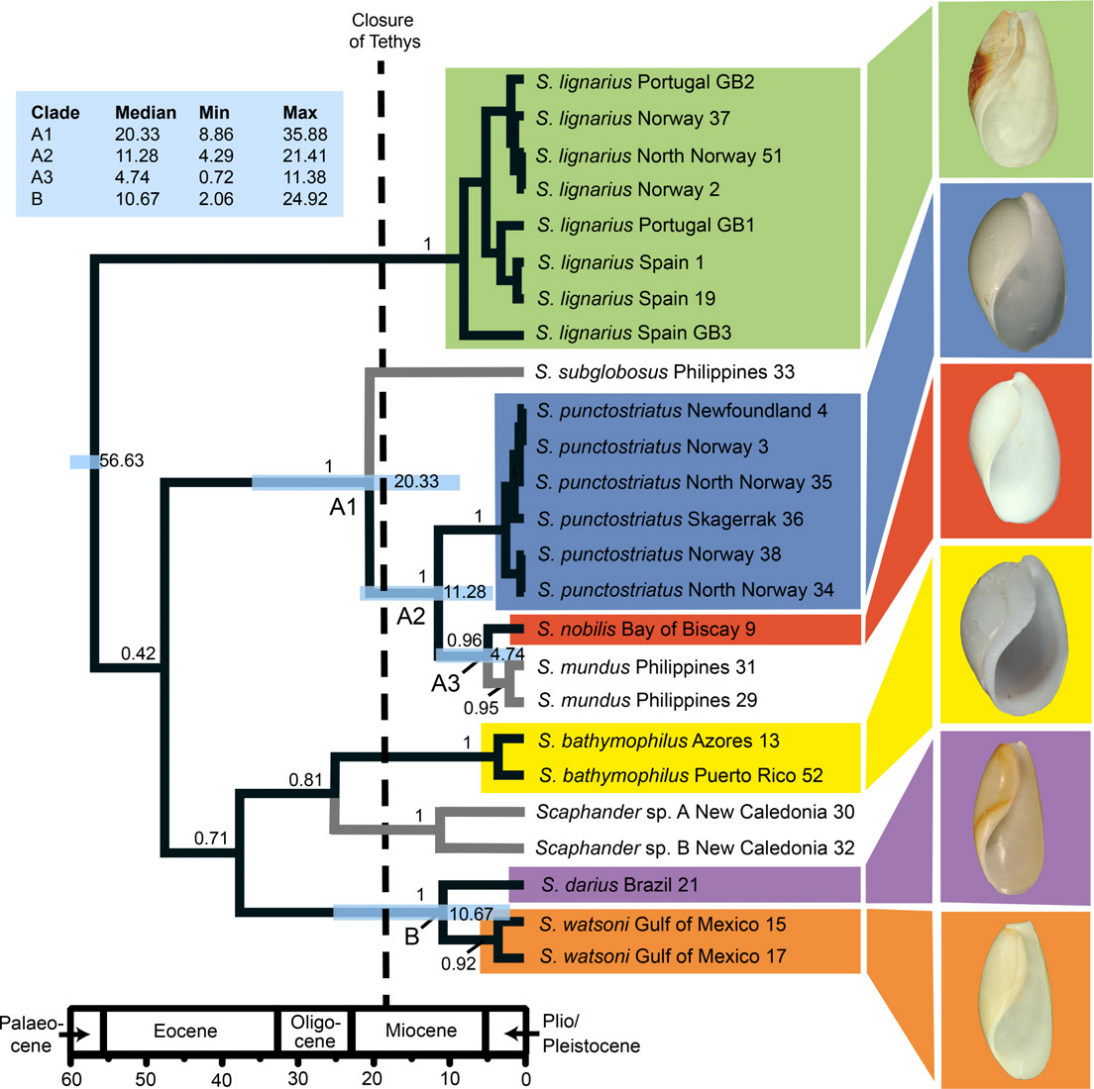
Chronogram produced by time‐calibrated Bayesian analysis of the concatenated three marker dataset (*COI* + *16S* + *28S*), using a relaxed molecular clock in BEAST). Branch labels show posterior probabilities, node labels show median ages of supported nodes and node bars represent 95% highest posterior density intervals (HPD). Median divergence times for nodes A1, A2, A3 and B in millions of years ago (Ma) are listed in the blue box with upper and lower limits of 95% HPD. The outgroup was pruned from the tree for clarity. The shells of the Atlantic species are illustrated (taken from Eilertsen & Malaquias, 2013a).

The individual gene trees were not in conflict (see Appendix S3), and the topology of the concatenated tree is consistent with the first phylogenetic analysis of *Scaphander* (Eilertsen & Malaquias, 2013a), with all morphological species forming monophyletic groups (Fig. 1). *Scaphander lignarius* is basal to the other species with high support, but the remaining deep divergences are not resolved [PP (posterior probabilities) < 0.9]. We focus on the species relationships within the genus that are well supported: clade A, with *S. subglobosus* (West Pacific) sister to a group containing *S. punctostriatus* (Atlantic) + *S. mundus* (WP) + *S. nobilis* (A), and clade B consisting of two West Atlantic sister species: *S. darius* and *S. watsoni* (Fig. 1).

### Divergence times and rates of evolution

The estimated age of divergence for node A1 representing the split between the West Pacific species *S. subglobosus* and clade A2, containing *S. punctostriatus* (amphi‐Atlantic), *S. nobilis* (amphi‐Atlantic), and *S. mundus* (IWP) is 20.33 Ma [highest posterior density (HPD) 35.88–8.86 Ma]. Node A2, the divergence between *S. punctostriatus* and the ancestral lineage of *S. nobilis* and *S. mundus,* is estimated at 11.28 Ma (HPD 21.41–4.29 Ma). The more recent split between *S. nobilis* and *S. mundus* (A3) is estimated at 4.74 Ma (HPD 11.38–0.72 Ma), and the divergence between the western Atlantic species *S. darius* and *S. watsoni* (clade B) is estimated to 13.16 Ma (HPD 27.02–2.4 Ma; see Fig. 1).

Average rates of substitution were highest for *COI* with 0.19% Myr^−1^, followed by *16S* with 0.11% Myr^−1^ and *28S* with 0.03% Myr^−1^. These rates are in line with those found for other gastropods under similar methodological approaches (Williams & Reid, 2004; Frey & Vermeij, 2008; Malaquias & Reid, 2009).

### Geographical and bathymetric distributions

**Figure 2:**
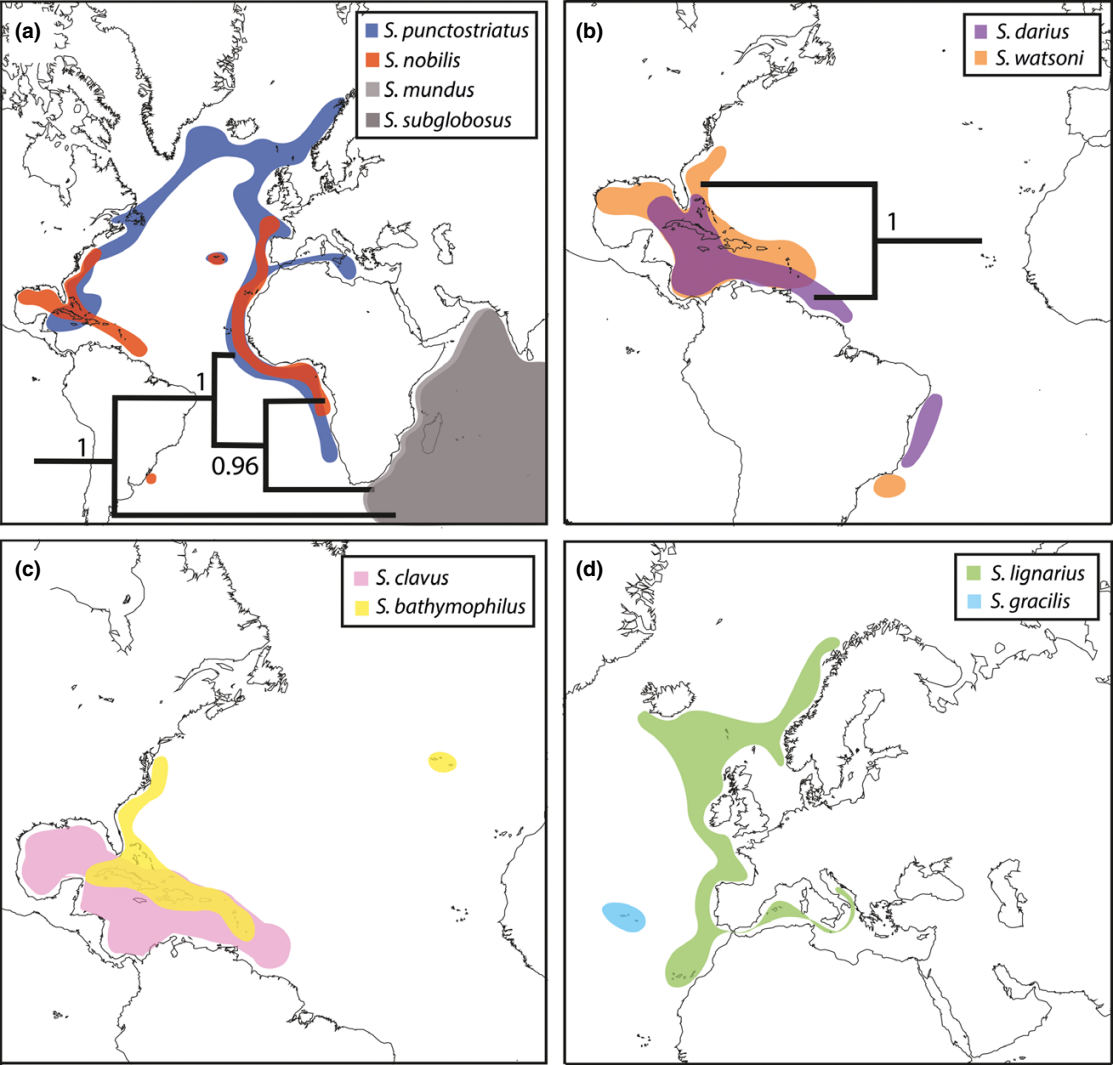
Geographical distributions and phylogenetic relationships of Atlantic *Scaphander* species. (a) clade A with species *S. subglobosus*,* S. punctostriatus*,* S. mundus* and *S. nobilis*; (b) clade B with species *S. watsoni* and *S. darius*; (c) *S. clavus* and *S. bathymophilus*; (d) *S. lignarius* and *S. gracilis*. Detailed maps and references to the literature surveyed for each species can be found in Eilertsen & Malaquias (2013a).

Two of the eight valid Atlantic species of *Scaphander* are amphi‐Atlantic (*S. nobilis* and *S. punctostriatus*), whereas one species, *S. bathymophilus*, is known from the western Atlantic and the mid‐Atlantic islands of the Azores (see Fig. 2). *Scaphander punctostriatus* and *S. bathymophilus* are represented in the molecular phylogeny by specimens from both east and west Atlantic (Table 1). For *S. nobilis*, only one specimen from the east Atlantic was suitable for molecular analysis, but specimens from the west Atlantic were studied and characters from the shell, digestive tract and reproductive system support that eastern and western populations are conspecific (Eilertsen & Malaquias, 2013a). Of the remaining Atlantic species, three are restricted to the western Atlantic (*S. darius*,* S. watsoni* and *S. clavus*), one is restricted to the eastern Atlantic (*S. lignarius*), and one is known only from the Azores (*S. gracilis*; Eilertsen & Malaquias, 2013a).

**Figure 3:**
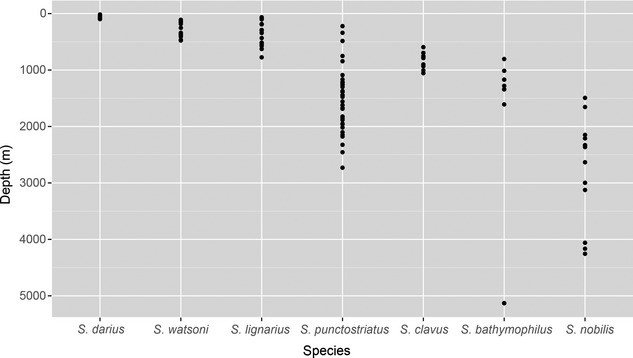
Depth distribution of Atlantic species of *Scaphander*, except *S. gracilis*, which is only known from shells. References to the literature surveyed for each species can be found in Eilertsen & Malaquias (2013a).

The bathymetric distributions of the Atlantic species of *Scaphander* are depicted in Fig. 3. The depth range of *S. gracilis* is not considered here because this species is only known from empty shells, which may be transported by currents or other animals. Three species are found on the continental shelf: *S. darius* has only been found between 16 m and 97 m, whereas the other two, *S. watsoni* and *S. lignarius*, occur at 110–476 m and 70–630 m, respectively. Two species have a mainly upper‐bathyal distribution (200–2000 m), namely *S. clavus* and *S. punctostriatus* found at 595–1056 m and 264–2730 m, respectively; but the latter also extends into the lower bathyal zone (2000–4000 m). *Scaphander bathymophilus* and *S. nobilis* have a wide bathyal to abyssal distribution with a bathymetric range of 805–5130 m and 1493–4255 m, respectively.

## Discussion

### Phylogeny, speciation and patterns of diversity in *Scaphander*

The monophyly of *Scaphander* has previously been confirmed by Eilertsen & Malaquias (2013a). That analysis showed that the Atlantic species‐group is not monophyletic, with several sister relationships between Atlantic and Indo‐Pacific lineages. Additionally, the systematics of the genus in the Atlantic was revised and species were delimited based on a combination of morpho‐anatomical characters and reciprocal monophyly of lineages rendered in a multilocus molecular phylogenetic analysis, which we have expanded here (Fig. 1). Eilertsen & Malaquias (2013a) found pronounced character displacement between the Atlantic species of *Scaphander*, particularly on reproductive structures, which, combined with shell shape, makes species identification reliable.

Only two Atlantic species of *Scaphander* were unavailable for molecular analysis: *S. gracilis*, known only from shells collected in the Azores; and *S. clavus*, from the west Atlantic (Eilertsen & Malaquias, 2013a). We acknowledge that incomplete taxon sampling can hamper inference of sister relationships and estimates of divergence times (Heath *et al*., 2008), but the present dataset remains one of the most complete datasets of a clade of deep‐sea invertebrates to have been assembled and analysed in a molecular phylogenetic context (but see Puillandre *et al*., 2010; Cabezas *et al*., 2012).

The combination of phylogenetic relationships and geographical distributions of species revealed little geographical overlap between sister taxa or clades (Fig. 2), which supports the prevailing view that allopatric speciation is dominant not only in shallow marine taxa (Meyer, 2003; Williams & Reid, 2004; Malaquias & Reid, 2009; Frey, 2010), but also in the deep sea. However, the overlap between the distributions of the western Atlantic sister species *S. darius* and *S. watsoni,* estimated to have diverged in the late Oligocene–Pliocene (median 10.67 Ma, HPD 24.92–2.06 Ma), hints at a possible case of sympatric speciation in *Scaphander*. Sympatric sister species are often characterized by ecological differentiation (e.g. Bolnick & Fitzpatrick, 2007; Frey, 2010), but no differences in bathymetric distribution, trophic ecology or habitat between these species were recognized, suggesting a similar ecological niche. We cannot, however, rule out the hypothesis of allopatric speciation followed by later geographical range shifts and secondary contact (Collin, 2003; Frey, 2010). Even though these two species are located on the same side of the Isthmus of Panama, speciation could be related to the uplift of the isthmus, which led to changes in current flow, salinity, temperature and primary production in the Atlantic and eastern Pacific, generating opportunities for transient allopatry and speciation (Williams & Reid, 2004; Lessios, 2008; Miura *et al*., 2010).

A pulse of diversification centred in the Oligocene and Miocene epochs has been suggested for shallow‐water organisms (e.g. Williams & Duda, 2008; Malaquias & Reid, 2009), and this has been also hypothesized more recently for deep‐sea fauna (Cabezas *et al*., 2012; Williams *et al*., 2013). An Oligocene/Miocene pulse of diversification is corroborated by the present results, in which median divergence time estimates of speciation events ranged between 26.64 and 7.38 Ma (Fig. 1).

There is a cline in diversity between the bathyal and abyssal zones with all but one *Scaphander* species (*S. darius*) present in the former bathymetric zone and only two (*S. nobilis* and *S. bathymophilus*) extending their range into the abyssal plains (> 4000 m; Fig. 3).

### Tethyan vicariance and deep‐sea dispersal across oceans

The presence of sister relationships between species and clades from the Atlantic and IWP dating from the Eocene–Miocene (nodes A1 and A2, with two Atlantic and two IWP species; Fig. 1) may suggest vicariance associated with the closure of the Tethyan Seaway in the early Miocene (*c*.  18–19 Ma; Rögl, 1998). Diversification events that resulted from Tethyan vicariance have been widely documented for shallow‐water organisms, and both molecular estimates and the fossil record indicate that differentiation between the biogeographical regions of the proto‐Mediterranean and the proto‐IWP region was already present in the Oligocene (e.g. Williams & Reid, 2004; Harzhauser *et al*., 2007; Frey & Vermeij, 2008; Malaquias & Reid, 2009; Cowman & Bellwood, 2013).

It is to be expected that deep‐sea organisms may have been affected earlier by the closure of the Tethyan Seaway than organisms associated with shallow habitats, but it is not clear if this is the case in *Scaphander*. The age estimates for the cladogenetic events spanning the Tethys closure are equivocal (nodes A1 and A2 in Fig. 1; A1, median 20.33 Ma, HPD 35.88–8.86 Ma; A2, median 11.28 Ma, HPD 21.41–4.29 Ma) and speciation could therefore have been driven either by Tethyan vicariance or by post‐Tethyan dispersal between the Atlantic and the Indo‐Pacific. On the other hand, the split between the Indo‐West Pacific *S. mundus* and the Atlantic *S. nobilis* (median 4.74 Ma, HPD 11.38–0.72 Ma), seems too recent to be associated with Tethyan vicariance, suggesting dispersal between the two ocean basins followed by subsequent isolation and allopatric speciation.

The shortest route of dispersal between the present‐day distributions of these two species is around the Cape of Good Hope (Fig. 2a). The southern tip of South Africa is a well‐known biogeographical barrier for shallow‐water tropical/temperate taxa because of the Benguela cold‐water system, which became established during the late Miocene (Siesser, 1980; Marlow *et al*., 2000; Teske *et al*., 2011). Nevertheless, the fossil record and molecular phylogenetics studies have documented successful dispersal and speciation events from the IWP to the Atlantic around South Africa in shallow‐water taxa after the establishment of the Benguela current system during the Plio‐Pleistocene (Vermeij & Rosenberg, 1993; Vermeij & Snyder, 2003; Rocha *et al*., 2005; Levy *et al*., 2013). These dispersal events have been attributed to changes in climate associated with the Plio‐Pleistocene glacial–interglacial cycles, which prompted modifications in ocean currents and water temperature (Vermeij & Rosenberg, 1993; Rocha *et al*., 2005).

The impact of these processes on the diversification of deep‐sea faunas is poorly understood, because of a general lack of knowledge about the distribution of deep‐sea organisms and the paucity of available phylogenetic hypotheses. Nevertheless, the lack of *Scaphander* species shared between the Atlantic and Indo‐Pacific realms suggests that barriers to dispersal are also present in the deep sea in this region. Evidence from deep‐sea foraminiferan assemblages and geochemistry suggest that the influx of fresh water caused by melting ice‐caps during the Plio‐Pleistocene strongly disrupted the deep‐sea current system in the area (e.g. Schnitker, 1974; Hodell *et al*., 1985; Gupta & Srinivasan, 1990), and this could have created opportunities for deep‐sea organisms to disperse.

Alternative dispersal routes between the Atlantic and the Indo‐Pacific are the Drake Passage in the southern Atlantic (opened in the late Oligocene; Beu *et al*., 1997), the Central American corridor before the final closure of the Isthmus of Panama (by the middle Miocene, there was a corridor about 2000 m deep; Coates & Obando, 1996; Lessios, 2008), and the trans‐Arctic route during the Pliocene (Marincovich & Gladenkov, 1999). However, these would have implied dramatic range shifts (see Eilertsen & Malaquias, 2013a; Fig. 2) or regional extinctions (but Palaeocene fossils are known from California; Weaver, 1949; Schoellhamer *et al*., 1981). Dispersal across the Eastern Pacific Barrier is known to be insurmountable for the majority of marine invertebrates (Williams & Reid, 2004; Frey, 2010) and further evidence, including a better representation of the diversity of Indo‐Pacific species, is necessary to further test these alternative hypotheses.

### Trans‐Atlantic dispersal via abyssal plains and along Arctic slopes

Two species of *Scaphander* are amphi‐Atlantic (*S. nobilis* and *S. punctostriatus*) and a third is known from the western Atlantic and the mid‐Atlantic islands of the Azores (*S. bathymophilus*; see Fig. 2). The remaining five Atlantic *Scaphander* species (*S. lignarius*,* S. darius*,* S. watsoni*,* S. clavus* and *S. gracilis*) have more limited distributions, restricted to one side of the Atlantic or to the Azores (Fig. 2). This raises the question of how connectivity is maintained across these long distances. Nothing is known about the reproduction and larval development of *Scaphander*, but the life‐span of heterobranch planktotrophic larvae is usually 15–42 days (Schaefer, 1996). The journey across the Atlantic by larval drift, following one of the main trans‐Atlantic surface currents, is estimated to take 60–400 days (Scheltema, 1971). Deep‐sea currents are slow (2–20 cm s^−1^; Heezen *et al*., 1966) compared to surface currents (trans‐Atlantic surface currents 14–90 cm s^−1^; Scheltema, 1971), and deep‐sea currents do not usually reach higher velocities than surface currents even during benthic storms (15–40 cm s^−1^; Hollister & McCave, 1984; Hollister, 1993). This indicates that unless *Scaphander* has much greater than average dispersal potential, trans‐Atlantic dispersal is unlikely to maintain connectivity between the eastern and western populations.

Comparing geographical and bathymetric ranges, it becomes evident that the three broadly distributed species are those with deeper bathymetric distributions (Fig. 3), which supports the traditional view that deeper species have wider geographical ranges (McClain & Hardy, 2010). Two of these species (*S. bathymophilus* and *S. nobilis*) extend their bathymetric range into abyssal depths below 4000 m (Fig. 3). We speculate that if these species have reproducing populations scattered over the abyssal plains, gene flow could then be maintained without requiring long‐distance dispersal of larvae, but instead by dispersal between populations. There is unfortunately a scarcity of data from these regions, because deep‐sea sampling has been mostly focused on coastal areas, areas around islands, hydrothermal vents or other chemosynthetic habitats, whereas the open‐ocean abyssal plains have hardly been sampled (Ramirez‐Llodra *et al*., 2010).

The ‘source–sink’ hypothesis of Rex *et al*. (2005) suggests that abyssal populations are non‐reproducing and require larval supply from bathyal populations. The main argument for this hypothesis is the decline in nutrient input with increasing distance from the continental slope, but a patchy distribution of areas with sufficient food supply, as predicted by the ‘temporal mosaic’ hypothesis (Grassle & Sanders, 1973; Rex & Etter, 2010), could enable a dynamic mosaic of reproductive populations of *Scaphander*. In fact, food availability is unlikely to be a limiting factor in *Scaphander*, because these snails feed almost exclusively upon foraminiferans (Eilertsen & Malaquias, 2013b), which are among the most abundant inhabitants of the abyssal sea floor (Gooday *et al*., 1992).

An interesting case is the distribution of *S. bathymophilus*, which occurs at depths of 805–5130 m in the Caribbean Sea, along the coast of the US north to Cape Hatteras (North Carolina), and also in the Azores (Eilertsen & Malaquias, 2013a; Figs 2 & 3). The broad longitudinal distribution of this species, spanning the width of the western Atlantic basin can be explained analogously to *S. nobilis,* but its isolation in the western Atlantic could be a consequence of a barrier effect caused by the Mid‐Atlantic Ridge hindering eastward dispersal in this species. The role of the Mid‐Atlantic Ridge in the dispersal of bathyal and abyssal organisms across the Atlantic is, however, poorly understood (Mullineaux *et al*., 2002; Zardus *et al*., 2006; Etter *et al*., 2011).

*Scaphander punctostriatus*, the most widely distributed *Scaphander* species, has only been recorded down to 2730 m (Fig. 3), albeit at Arctic and sub‐Arctic latitudes, where the distance between the eastern and western margins is shorter, and the coasts of Iceland and Greenland could provide ‘staging‐posts’ for dispersal. In fact the percentage of amphi‐Atlantic species at Arctic and boreal latitudes has been documented to be higher than at temperate and tropical latitudes (Vermeij, 2005; García & Bertsch, 2009).

## Conclusions

The sister relationships between Atlantic and IWP lineages dating from the middle Eocene to late Miocene suggest vicariance events caused by the closure of the Tethyan Seaway, but they do not support a comparatively older diversification of deep‐sea faunas. However, our results corroborate the hypothesis of a pulse of diversification centred in the Oligocene and Miocene epochs. A post‐Tethyan divergence between Atlantic and IWP species is hypothesized to result from dispersal around South Africa during episodic disruptions of the deep‐sea regional current system caused by glacial–interglacial cycles. Allopatric speciation was prevalent, but one potential case of sympatric speciation was detected between two western Atlantic species. Amphi‐Atlantic species have comparatively deeper distributions (inhabiting bathyal‐abyssal depths), and we hypothesize that trans‐Atlantic dispersal is attained by connectivity between reproductive populations inhabiting the abyssal sea floor and by dispersal across the shelf and slope of Artic and sub‐Arctic regions.

## Biosketches

**Mari H. Eilertsen** is a PhD student at the Marine Biodiversity Research Group at the University of Bergen, Norway. Her main research interests are the biogeography and speciation of marine invertebrates in the deep sea. In addition to gastropods, her study groups include ampharetid polychaetes and calcareous sponges.

**Manuel António E. Malaquias** is an associate professor in invertebrate systematics at the Department of Natural History, University Museum of Bergen, University of Bergen, Norway. His main research interests are the diversity, systematics and phylogeny of cephalaspidean gastropods and the study of speciation and biogeography in the marine realm.

## Supporting information


**Appendix S1** gblocks settings.
**Appendix S2** Best‐fit model and estimated parameters for phylogenetic analysis.
**Appendix S3** Single gene chronograms.
