# Supplementary material for: Speciation in the dark: diversification and biogeography of the deep‐sea gastropod genus Scaphander in the Atlantic Ocean
Source: J Biogeogr. 2015 Jan 30;42(5):843–55. doi: 10.1111/jbi.12471 (PMC4964956; doi:10.1111/jbi.12471)

*Journal of Biogeography*

**Supporting information**

**Speciation in the dark: diversification and biogeography
of the deep-sea gastropod genus *Scaphander* in the Atlantic Ocean**

Mari H. Eilertsen and Manuel António E. Malaquias

**Appendix S1** Gblocks settings. Default settings in parentheses.

|  | *COI*-B | *16S* | *28S* |
| --- | --- | --- | --- |
| Minimum number of sequences for conserved positions | 13 | 13 | 11 |
| Minimum number of sequences for flank positions | 15 (20) | 15 (20) | 13 (17) |
| Maximum number of contigs at non-conserved positions | 10 (8) | 10 (8) | 10 (8) |
| Minimum length of block | 5 (10) | 5 (10) | 5 (10) |
| Allowed gap positions | all (none) | all (none) | all (none) |
| Original number of positions | 461 | 459 | 1510 |
| Number of positions in Gblocks alignment | 451 | 432 | 1485 |

**Appendix S2** Best-fitting models and estimated parameters for phylogenetic analysis.

| Parameter | *COI* | *16S* | *28S* |
| --- | --- | --- | --- |
| Number of specimens | 24 | 24 | 21 |
| Number of characters | 451 | 432 | 1485 |
| Best-fitting model | GTR+I+G | GTR+I+G | GTR+I |
| Freq. A | 0.3604 | 0.3197 | 0.1905 |
| Freq. C | 0.2351 | 0.1558 | 0.3242 |
| Freq. G | 0.2067 | 0.2165 | 0.2651 |
| Freq. T | 0.1979 | 0.3080 | 0.2203 |
| Gamma shape | 0.2340 | 0.5620 | — |
| Proportion of invariant sites | 0.6380 | 0.5740 | 0.8340 |
| R-matrix [A–C] | 425.4453 | 0.0285 | 1.3058 |
| R-matrix [A–G] | 45861.9973 | 406.0528 | 9.3099 |
| R-matrix [A–T] | 2685.75 | 225.8930 | 0.7026 |
| R-matrix [C–G] | 995.5860 | 0.0285 | 0.6109 |
| R-matrix [C–T] | 2951.4398 | 436.0289 | 0.9823 |
| R-matrix [G–T] | 1.0000 | 1.0000 | 1.0000 |

**Appendix S3** (overleaf) Single-gene chronograms produced by time-calibrated Bayesian analysis of the three single-marker datasets (a, *COI*; b, 16S rRNA; c, 28S rRNA), using a relaxed molecular clock in beast (Drummond & Rambaut, 2007) and calibrated with the first appearance of *Scaphander* in the fossil record (*c.* 57.5 Ma). Branch labels show posterior probabilities, scale bars in millions of years. The outgroup has been removed from the trees.


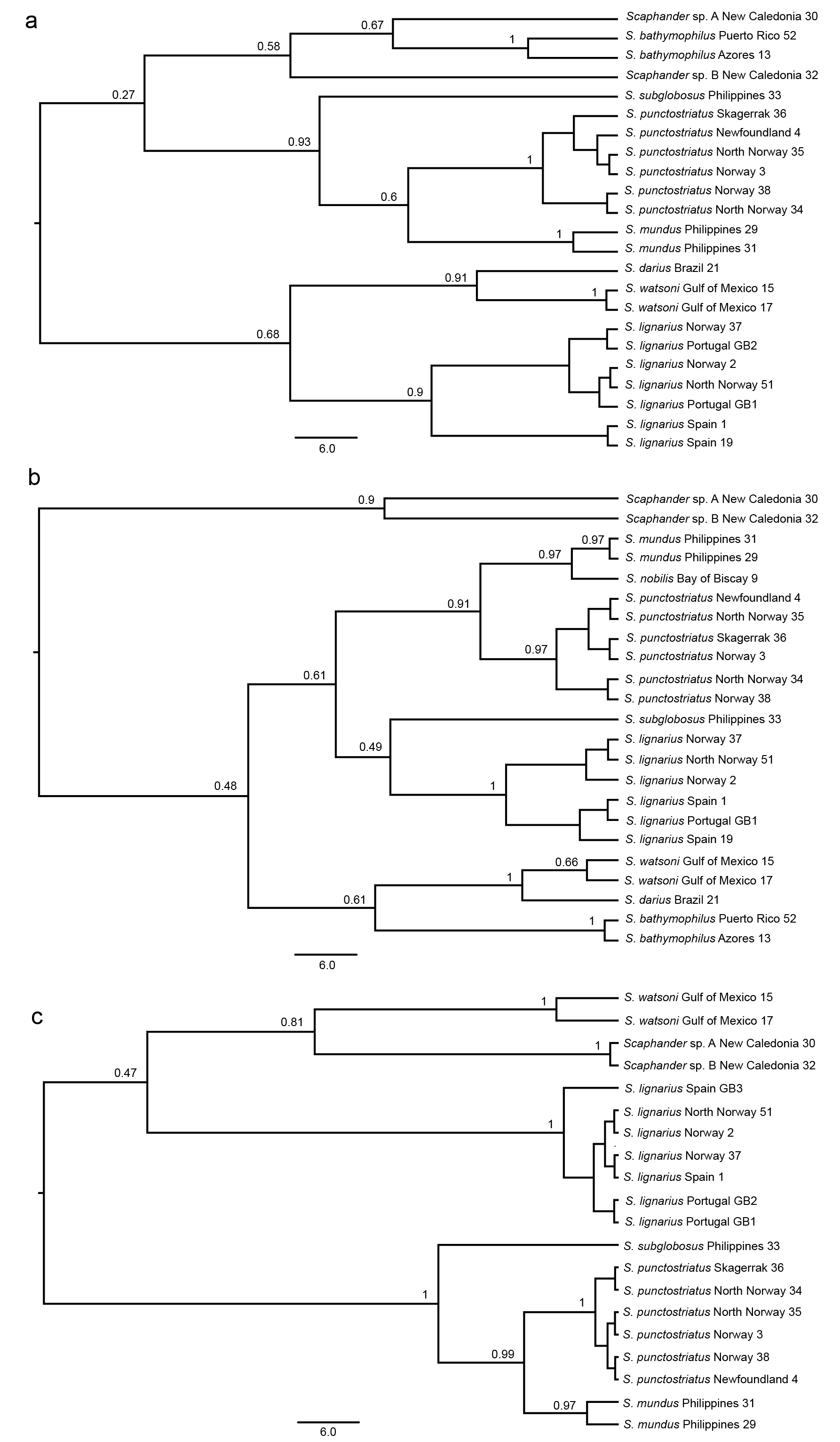

Supplement: Supplementary file 1 — Appendix S1 gblocks settings. Appendix S2 Best‐fit model and estimated parameters for phylogenetic analysis. Appendix S3 Single gene chronograms. [file JBI-42-843-s001.doc]
